# Biofabrication of novel silver and zinc oxide nanoparticles from *Fusarium solani* IOR 825 and their potential application in agriculture as biocontrol agents of phytopathogens, and seed germination and seedling growth promoters

**DOI:** 10.3389/fchem.2023.1235437

**Published:** 2023-08-04

**Authors:** Joanna Trzcińska-Wencel, Magdalena Wypij, Artur P. Terzyk, Mahendra Rai, Patrycja Golińska

**Affiliations:** ^1^ Department of Microbiology, Faculty of Biological and Veterinary Sciences, Nicolaus Copernicus University in Toruń, Toruń, Poland; ^2^ Physicochemistry of Carbon Materials Research Group, Department of Chemistry of Materials, Adsorption and Catalysis, Faculty of Chemistry, Nicolaus Copernicus University in Toruń, Toruń, Poland; ^3^ Nanobiotechnology Laboratory, Department of Biotechnology, SGB Amravati University, Amravati, India

**Keywords:** applied microbiology, biocontrol, biofabrication, biogenic nanoparticles, bionanotechnology, crop protection, mycosynthesis, plant pathogens

## Abstract

**Introduction:** Plant pathogenic microorganisms adversely affect the growth and yield of crops, which consequently leads to losses in food production. Metal-based nanoparticles (MNPs) can be a remedy to solve this problem.

**Methods:** Novel silver nanoparticles (AgNPs) and zinc oxide nanoparticles (ZnONPs) were biosynthesized from *Fusarium solani* IOR 825 and characterized using Dynamic Light Scattering (DLS), Fourier Transform Infrared (FTIR) spectroscopy, Transmission Electron Microscopy (TEM), X-ray diffraction (XRD) and measurement of Zeta potential. Antibacterial activity of NPs was evaluated against four plant pathogenic strains by determination of the minimum inhibitory (MIC) and biocidal concentrations (MBC). Micro-broth dilution method and poisoned food technique were used to assess antifungal activity of NPs against a set of plant pathogens. Effect of nanopriming with both types of MNPs on maize seed germination and seedlings growth was evaluated at a concentration range of 1–256 μg mL^-1^.

**Results:** Mycosynthesis of MNPs provided small (8.27 nm), spherical and stable (zeta potential of −17.08 mV) AgNPs with good crystallinity. Similarly, ZnONPs synthesized by using two different methods (ZnONPs(1) and ZnONPs(2)) were larger in size (117.79 and 175.12 nm, respectively) with Zeta potential at −9.39 and −21.81 mV, respectively. The FTIR spectra showed the functional groups (hydroxyl, amino, and carboxyl) of the capping molecules on the surface of MNPs. The values of MIC and MBC of AgNPs against bacteria ranged from 8 to 256 μg mL^-1^ and from 512 to 1024 μg mL^-1^, respectively. Both types of ZnONPs displayed antibacterial activity at 256–1024 μg mL^-1^ (MIC) and 512–2048 μg mL^-1^ (MBC), but in the concentration range tested, they revealed no activity against *Pectobacterium carotovorum*. Moreover, AgNPs and ZnONPs inhibited the mycelial growth of *Alternaria alternata, Fusarium culmorum, Fusarium oxysporum, Phoma lingam,* and *Sclerotinia sclerotiorum*. MIC and MFC values of AgNPs ranged from 16–128 and 16–2048 μg mL ^-1^, respectively. ZnONPs showed antifungal activity with MIC and MFC values of 128–2048 μg mL^-1^ and 256–2048 μg mL^-1^, respectively. The AgNPs at a concentration of ≥32 μg mL^-1^ revealed sterilization effect on maize seeds while ZnONPs demonstrated stimulatory effect on seedlings growth at concentrations of ≥16 μg mL^-1^ by improving the fresh and dry biomass production by 24% and 18%–19%, respectively.

**Discussion:** AgNPs and ZnONPs mycosynthesized from *F. solani* IOR 825 could be applied in agriculture to prevent the spread of pathogens. However, further toxicity assays should be performed before field evaluation. In view of the potential of ZnONPs to stimulate plant growth, they could be crucial in increasing crop production from the perspective of current food assurance problems.

## 1 Introduction

Plant diseases caused by pathogenic microorganisms adversely affect the growth and yield of crops, which consequently leads to losses in food production (Peng et al., 2021). Recently, Van Dijk et al. (2021) presented a set of projections on the growth of food demand between 2010 and 2050 and the risks of food shortage and hunger. The majority of food safety research focuses on common denominators such as climate change, resistant pathogens, and the need for sustainable agriculture while addressing human health and environmental concerns (Zakirov et al., 2021). The use of technological advances, including mechanization, pesticides or fertilizers, allowed to achieve higher productivity of crop plants (Jacquet et al., 2022). It is estimated that 70%–80% of plant diseases are caused by fungal pathogens particularly by secretion of toxins that cause various physiological disorders, growth inhibition, chlorosis, wilting, etc. (Peng et al., 2021). Maize belongs to the most frequent crop plants worldwide, including Poland (Król et al., 2018), and is particularly vulnerable to pathogenic fungi, e.g., *Fusarium* spp*.* (Czembor et al., 2015). In addition, microbial diseases lead to yield losses, while mycotoxins produced by fungi contaminate and reduce the quality of maize grains (Czembor et al., 2015). A number of synthetic chemical pesticides are required to prevent crop diseases. However, some negative aspects of pesticides on the environment and human health have been unequivocally documented, therefore limiting their use is of high priority (Jacquet et al., 2022). A number of studies have been conducted to design the remedy for addressing this excessive and seemingly irreplaceable application of traditional chemicals which showed that a prospective solution may be the use of products at the nanoscale (Pestovsky and Martinez-Antonio, 2017). Metal-based nanoparticles (MNPs) show promising potential for use in agriculture field as they demonstrate broad range of antibacterial and antifungal activities (Singh et al., 2021). The use of bio-nanoparticles (bio-NPs) as antimicrobial agents might empower a significant reduction in the dose used while maintaining antimicrobial efficacy compared to conventional materials. From an agro-ecological point of view, the application of nanoparticles (NPs) obtained from biological sources may mitigate the negative consequences of agriculture procedures imposed on the environment (Hazarika et al., 2022).

To date, various studies on the potential use of MNPs as fungicides have been reported (Malandrakis et al., 2022). Silver and zinc oxide nanoparticles (AgNPS and ZnONPs, respectively) display antimicrobial activity depending, in particular, on their physicochemical properties and the targeted pathogen (Gudkov et al., 2021). Some reports showed antimicrobial activity of biosynthesized AgNPs and ZnONPs against, e.g., *Escherichia coli, Pseudomonas aeruginosa*, *Staphylococcus aureus, Xanthomonas oryzae* pv. *oryzae, Alternaria alternata, Pyricularia oryzae, Sclerotinia sclerotiorum,* and many more (Consolo et al., 2020; El Sayed and El-Sayed, 2020; Ogunyemi et al., 2020; Pillai et al., 2020). It is suggested that the high ability of biosynthesized NPs to control microbial pathogens is due to their large surface area, small size, and high concentration of released ions or capping agents on the surface of the metallic core (Gudkov et al., 2021). In addition, zinc plays an important role in cell function and proliferation (membrane stability, metabolic processes, enzymatic activity, photosynthesis) and consequently in plant physiology (Cakmak et al., 2010; Faizan et al., 2020). It has been reported that the foliar and soil use of ZnONPs for maize biofortification resulted in higher Zn availability and uptake and affected Zn content in edible crop organs than the commonly used zinc sulfate (ZnSO_4_). The higher bioavailability of zinc in nano form is attributed to its lower solubility than that of ZnSO_4_ salts (Umar et al., 2021). In another study, maize and wheat supplementation with ZnONPs in lower doses (100 mg L^-1^) improved seedling length and biomass production, and enzyme activity (α-amlyase, antioxidative system enzymes), which suggests potential application of ZnONPs as stimulators of crop plant growth (Srivastav et al., 2021). However, some phytotoxic effects of MNPs to plant development has been summarized by Abedi et al. (2022). This aspect is important before the evaluation of plant growth protection and/or promotion with the use of nanoparticles in the field. The diverse properties (small sizes, variable shapes, chemical nature) of MNPs determine their high reactivity and subsequently their uptake, translocation, and interactions in plants (de la Rosa et al., 2021). Some studies reported, that MNPs affect basic cellular processes by inducing oxidative stress, disrupting cell membrane transport or altering gene expression (Lee et al., 2013; Akhtar et al., 2022). Recently, Wan and coworkers (2019) found that ZnONPs (at concentration >100 mg L^-1^) caused endocytosis and led to the rearrangement of microfilament in the epidermal cells of elongation zones of *Arabidopsis* seedlings. However, above changes were temporary, and plants after NPs-related stress recovered faster than those treated with Zn^2+^ ions. In addition, MNPs introduced into the soil environment may cause changes in soil fertility, as well as affect microorganisms and invertebrates. Wei and coworkers (2021) reported diversified effects of ZnO, Cu, and **γ-**Fe_2_O_3_ NPs on plant (*Salvia miltiorrhiza*) growth and soil environment. For instance, the effect of NPs on seedling aboveground biomass depended on the type of NPs and dose applied (100 and 700 mg kg^-1^). CuNPs showed no effect on plant biomass production at tested concentrations. In the case of ZnONPs, their lower concentrations (100 mg kg^-1^) promoted growth, while higher concentrations (700 mg kg^-1^) reduced it. In turn, **γ-**Fe_2_O_3_ NPs at both tested concentrations promoted plant growth. Moreover, the increase in the relative abundance of *S. miltiorrhiza* rhizosphere microorganisms, namely, the plant growth-promoting bacteria (*Sphingomonas*), superoxide dismutase producers (*Aminobacter*), and the metal-tolerant bacteria (*Thiobacillus* and *Metarhizium*) after MNPs-treatment was observed (Wei et al., 2021). The above findings indicate that various nanomaterials introduced into the environment might have undesirable effects, thus there is an urgent need to properly identify and study the effects of plant exposure to nanoparticles.

The synthesis of nanoparticles covers a variety of approaches, which include physical, chemical, biological, and their combination (Rai et al., 2021). The formation and structural parameters of nanoparticles depend on the reaction conditions and type of the stabilizing agent used in the synthesis (Lallo da Silva et al., 2019). Numerous studies have shown that the ability to form nanoparticles is demonstrated by plants (Balachandar et al., 2019; Verma and Bharadvaja, 2022), bacteria (Quinteros et al., 2019; Saeed et al., 2020), actinomycetes (Wypij et al., 2021, 2022), fungi (Feroze et al., 2020), and viruses (Le et al., 2017) by using their live cultures, biomass, extracts, and metabolites. Mycosynthesis of various nanoparticles is intensively studied by many researchers (Ingle et al., 2009; Abd El-Aziz et al., 2015; Clarance et al., 2020). The benefits of fungal-mediated synthesis of nanoparticles include the simplicity of preparation and the relatively undemanding stages of the synthesis process (Ganachari et al., 2012; Zare et al., 2017). Moreover, these microorganisms seem to possess an outstanding ability to tolerate metals, and fungal metabolites are involved in the reduction of metal salts as well as the further formation of metallic nanoparticles (Kobashigawa et al., 2019; Rai et al., 2021). Further research needs to focus on optimizing the synthesis process to obtain efficient scale production and nanoproducts with the sought-after bioactivity important for their potential application (Sasani et al., 2023). The adaptation of environmental conditions to the growth of microorganisms as well as synthesis conditions assists in the efficient production of nanoparticles with well-defined morphology and biological activity (Paul and Roychoudhury, 2021; Murillo-Rábago et al., 2022).

The aim of the study was to evaluate the ability of fungal extract from *F. solani* IOR 825 to synthesize AgNPs and ZnONPs and to optimize the reaction parameters for high production yields and biological activity of generated nanoproducts. It is the first report on the synthesis of silver and zinc oxide nanoparticles from *Fusarium solani* IOR 825 strain. The novel nanoparticles were assessed for antimicrobial activities against a wide set of phytopathogenic bacteria, fungi, and oomycetes. Moreover, the potential of these AgNPs and ZnONPs for sterilization of maize (*Zea mays*) seeds and the effect of seeds nanopriming on their germination and seedling growth (shoot and root elongation, fresh and dry mass production) was also evaluated. This is also the first report on the potential use of nanoparticles from *F. solani* species in agriculture for the protection and promotion of maize growth.

## 2 Material and methods

### 2.1 Biosynthesis and determination of physical and chemical properties of metal-based nanoparticles

#### 2.1.1 Optimization of fungal growth

For the synthesis of AgNPs and ZnONPs fungal extracts obtained from *F. solani* IOR 825 isolated from parsley was used. The fungal strain was purchased from the bank of plant pathogens of the Institute of Plant Protection (IOR), the National Research Institute of Poland. In order to optimize efficient production of fungal biomass, each strain was cultured in three kinds of media, namely, potato dextrose broth (PDB, A&A Biotechnology), Sabouraud dextrose broth (SDB, Becton Dickinson) and Czapek dox (CDB, Oxoid). Broths (200 mL) were inoculated with the disc (Ø 5 mm) of fungal strain grown for 7 days at 26°C on potato dextrose agar (PDA, Becton Dickinson) cut with a sterile cork borer. Inoculated broth was incubated for 10 days at 26°C in shaking conditions at 150 rpm. Next, fungal biomass was harvested at 6000 rpm for 10 min and washed three times with sterile distilled water to remove medium components. Obtained biomass was weighed and the fungal culture medium that promoted the most efficient biomass production was selected for further studies on mycosynthesis of nanoparticles.

#### 2.1.2 Preparation of fungal extract and mycosynthesis of nanoparticles

The fungal extract used for the synthesis of silver and zinc oxide nanoparticles was prepared from the fungal biomass, as described previously by Wypij et al. (2022) and Trzcińska-Wencel et al. (2023).

Synthesis of AgNPs was carried out as described previously by Trzcińska-Wencel et al. (2023).

Zinc oxide nanoparticles were synthesized after challenging the fungal extract with the aqueous solution of ZnSO_4_ (100 mM), as a precursor. Two methods were developed for sufficient synthesis of nanoparticles. The first synthesis method (1) involved the combination of the fungal extract, ZnSO_4,_ and NaOH in a ratio of 1:1:1 (v/v/v) with a final volume of 150 mL and heating for 15 min at 40°C. In the second method (2), the pH of the mixture of fungal extract and ZnSO_4_ (1:1, v/v) was adjusted at pH 11 with NaOH (0.4 M). Finally, biosynthesized ZnONPs were centrifugated at 6000 x g for 10 min (Thermo Scientific, United States), washed three times with sterile distilled water to remove unwanted components, and obtained pellet was dried at 37°C.

#### 2.1.3 Detection and characterization of NPs

Confirmation of the NPs synthesis and evaluation of the physico-chemical properties of the nanoparticles were carried out as previously described by Wypij et al. (2021). The formation of NPs was verified using UV-Vis spectrometry (NanoDrop One, Thermo Fisher Scientific, United States) in the wavelength range from 200 to 700 nm at the resolution of 1 nm.

*Transmission Electron Microscopy (TEM) and Energy Dispersive X-ray Spectroscopy (EDX)* The morphology, size, and elemental composition of the NPs were specified by TEM and EDX analysis using a transmission electron microscope coupled with energy dispersive X-ray spectrometer (FEI Tecnai F20 X-Twintool, Fei, Hillsboro, OR, United States). Sample preparation involved the suspension of NPs in deionized water and deposition of the solution (2 µL) on a carbon-coated copper grid (mesh size 400 µm). Samples were dried at room temperature for 24 h prior to measurements.

*X-ray diffraction (XRD)* The powder of NPs was deposited on the sample holder acquiring a smooth surface and used for XRD studies. Analysis was performed with X’ Pert PRO Analytical X6 diffractometer (PANalytical, Netherlands) with Ni filter and CuKα (λ = 1.54056 Å) radiation source. The diffraction was recorded over a 2θ range of 5°–120° and compared with the standard database.

*Fourier Transform Infrared Spectroscopy (FT-IR)* For FTIR analysis, the potassium bromide (KBr) method was used, briefly dried NPs were ground with KBr (1:1, w/w) and used for measurements. The spectrum was recorded in the range of 4,000–400 cm^-1^ using a spectrometer (Spectrum 2000; Perkin-Elmer, Waltham, Massachusetts, United States) running at the resolution of 4 cm^-1^.

*Dynamic light scattering (DLS)* Dynamic light scattering and zeta potential measurement were used for the determination of size distribution (hydrodynamic diameter) and stability (zeta potential value) of biosynthesized NPs. Dried NPs were suspended in ultrapure Milli-Q water and sonicated for 15 min at 30 kHz prior to measurements. The analysis was performed using Particulate Systems, NanoPlus HD (Micromeritics, Particulate Systems, Norcross, GA, United States).

### 2.2 Antimicrobial activity studies

#### 2.2.1 Antibacterial activity

The antibacterial activity of synthesized MNPs was assessed against plant pathogenic bacterial strains, namely, *Agrobacterium tumefaciens* IOR 911, *Pectobacterium carotovorum* PCM 2056, *Pseudomonas syringae* IOR 2188 and *Xanthomonas campestris* IOR 512 according to Clinical Laboratory Standard Institute (CLSI, 2012). Strains were grown in 20 mL of trypticase soy broth (TSB, Becton Dickinson) for 24 h at 26°C under shaking conditions (120 rpm) and used for the preparation of inocula in distilled water at an optical density of 0.5 McFarland scale (1.5 × 10^8^ CFU mL^-1^). Minimal inhibitory concentrations (MICs) of nanoparticles were determined, in triplicate, by the 2-fold microdilution method in 96-well plates at the concentration range 1–2048 μg mL^-1^. The final concentration of bacteria in each well was 1.5 × 10^−6^ CFU mL^-1^ while the final volume of sample in the wells was 150 µL. Both a negative control (sterile medium) and a positive control (inoculated medium) were performed. The MICs of NPs were defined as the concentrations for which no visible growth was noted after 24 h of incubation at 26°C.

After the determination of MICs of NPs, an aliquot (100 µL) of samples from wells with no visible bacterial growth was spread on tryptic soy agar (TSA, Becton Dickinson) in Petri plates and incubated for 24 h at 26°C. The lowest concentration of NPs which resulted in the elimination of 99.9% of bacteria was identified as minimal biocidal concentration (MBC).

### 2.3 Antifungal activity

#### 2.3.1 Tested microorganisms

Antifungal activity of MNPs was evaluated against: *Alternaria alternata*, *Alternaria alternata* IOR 1783, *Aspergillus niger*, *Botrytis cinerea* IOR 1873, *Colletotrichum acutatum* IOR 2153, *Fusarium culmorum*, *Fusarium culmorum* IOR 2333, *Fusarium culmorum* DSM 114849, *Fusarium graminearum* A, *Fusarium graminearum* D, *Fusarium oxysporum*, *Fusarium oxysporum* IOR 342, *Fusarium oxysporum* D, *Fusarium poae* A, *Fusarium tricinctum*, *Penicillium* sp., *Penicillium spinulosum*, *Phoma lingam* IOR 2284, *Sclerotinia sclerotiorum* IOR 2242 and oomycetes *Phytophthora megasperma* IOR 404, *Phytophthora cryptogea* IOR 2080 and *Phytophthora plurivora* IOR 2303.

#### 2.3.2 Inhibition of fungal mycelia growth

Antifungal activity of nanoparticles was evaluated, in triplicate, using poisoned food technique. The final NPs concentrations in the PDA medium were 100 and 200 μg mL^-1^ for AgNPs, and 100 and 1000 *** *** μg mL^-1^ for ZnONPs. The aqueous stock solution of nanoparticles was used to prepare the final concentration of NPs in the agar medium. The required stock solution of nanoparticles was added into cooled molten PDA (45°C) followed by manual rotation in a sterile Erlenmeyer flask to disperse the NPs in the medium. The medium (20 mL) was dispensed into sterile Petri dishes (9 cm in diameter) with a sterile serological pipette to avoid bubbles. The medium was allowed to solidify at room temperature (23°C ± 2°C) for 1 hour. Agar discs with fungal mycelia (6 mm in diameter), grown on PDA medium for 7 days, were cut using a sterile cork borer and aseptically inoculated at the center of the Petri plates. Control plates were PDA media without the nanoparticles inoculated following the same procedure. The plates were incubated at 28°C. The fungal colony diameter was recorded after 7 days of incubation. The percentage inhibition of the mycelial growth of the test fungi by the nanoparticles was calculated using the formula by Philippe et al. (2012).

$$Inhibition\;of\;mycelial\;growth\;\left(MGI\right)\;\left(\%\right)=\frac{dc-dct}{dc}\;x100$$

where dc is the mean diameter of the colony in the control sample, and dct is the mean diameter of the colony in the treated sample.

#### 2.3.3 Inhibition of spore germination

To assess the ability of NPs to inhibit spore germination, minimum inhibitory and minimum fungicidal concentrations (MICs and MFCs) were determined according to the Clinical Laboratory Standard Institute (CLSI, 2012) protocol with slight modifications. Spore suspensions were prepared by washing fungal colonies grown on PDA medium for 14 days at 26°C with 5 mL sterile distilled water. The spore suspensions were then filtered through a sterile cotton wool filter to remove any residual mycelia. The density of spores in the suspension was set at approx. 1 × 10^6^ spores per mL by using a cell counting chamber (Brand, Germany), 10-fold diluted and used for assays. Assay was performed as earlier described for bacteria, albeit in potato dextrose broth (PDB). The final concentration of spores in each well was 1 × 10^3^ spores per mL. Sterile broth provided negative control while inoculated with spores was positive control. Inoculated plates were incubated for 2 days at 26°C and recorded for MIC. Finally, aliquots (100 µL) from wells without visible microbial growth were spread on the PDA surface and incubated under the same conditions for 7 days for MFC determination. MFC was defined as the lowest concentration at which no fungal growth was observed.

### 2.4 Influence of NPs on maize (*Zea* mays) seed germination and seedling growth

#### 2.4.1 Seeds sterilization and priming with NPs

For germination assays, seeds of maize were purchased from Torseed S.A (Toruń, Poland). Seeds were surface sterilized for 30 min with 30% H_2_O_2_ and 70% ethanol (1:1, v:v), followed by 5 times washing with sterile distilled water and placed on ½ Murashige and Skoog (MS) agar medium in sterile culture boxes for 10 days at 22°C ± 2°C for germination. In order to analyze sterilization efficiency, 100 µl of post-wash water from each variant was spread on sterile PDA and Reasoner’s 2A agar (R2A) media and incubated for 7 days for the detection of microbial contaminations.

Two varieties of experiments were performed. In the first one, the sterilization potential of both types of NPs to seed surface was evaluated. The 15 non-sterile seeds were soaked for 30 min with 25 mL of AgNPs or ZnONPs solution in water at concentrations of 1, 8, 32, 64, 128, and 256 μg mL^-1^, washed 5 times with sterile distilled water. Seed sterilized by the standard method and soaked with sterile distilled water served as the control. All seeds were placed on ½ Murashige and Skoog (MS) agar medium in sterile Petri plates for 3 days at 22°C ± 2°C for germination. The germinated seeds were transferred into culture boxes with ½ MS and grown for another 7 days for seedling growth.

As treatment with ZnONPs did not result in seed sterilization, in the second variant of the experiment, seeds treatment with ZnONPs at a concentration range of 1–256 μg mL^-1^ was preceded by standard sterilization method to determine effect of nanoparticles on seed germination and seedling growth. In both experiments seeds treated with sterile distilled water were implemented as a control, and all experiments were performed in triplicate.

#### 2.4.2 Estimation of germination and growth parameters

The root and shoot length were measured with the ruler in centimeters [cm]. Fresh and dry weight mass were also determined in miligrams [mg]. Various parameters of germination and seedlings growth were calculated using the following formulas:

$$Germination\;percentage\;\left(\%\right)=\left(\sum n/N\right)\;x\;100$$

where ∑n–the total number of seeds germinated after 10 days; N is the total number of seeds used for analysis. Scott et al. (1984)

$$Mean\;germination\;time\;\left(MGT\right)=\Sigma \;f*x/\Sigma n$$

where f –number of germinated seeds at day x; x - number of day from sowing; Σn–total number of germinated seeds. Orchard (1997)

$$Germination\;Rate\;Index\;\left(GRI\right)=G1/1+G2/2+\ldots +Gx/x$$

where G1, G2 … Gx–germination percentage in the subsequent days after sowing. Esechie (1994)

Vigour index I=Germination %×SL

where SL - Seedling length (Root + Shoot). Abdul-Baki and Anderson (1973)

Vigour index II=Germination %×SDW

where SDW - Seedling dry weight (Root + Shoot)

### 2.5 Statistical analyses

Statistica software (StatSoft Inc., Tulsa, OK, United States States) was used for data analysis. Results were shown as mean ± standard error (SE). The means were then compared to determine statistical significance (if *p* < 0.05) by One-way ANOVA followed by Tukey’s test.

## 3 Results

### 3.1 Biosynthesis and determination of physical and chemical properties of metal-based nanoparticles

#### 3.1.1 Optimization of fungal growth

The most efficient growth of *Fusarium solani* IOR 825 was observed in SDB medium (42.75 ± 1.58 g L^-1^), followed by CDB (34.22 ± 1.21 g L^-1^) and PDB (20.44 ± 1.03 g L^-1^). For further studies, SDB was selected for *F. solani* IOR 825 biomass production.

#### 3.1.2 Mycosynthesis, visual detection, and characterization of NPs

**FIGURE 1:**
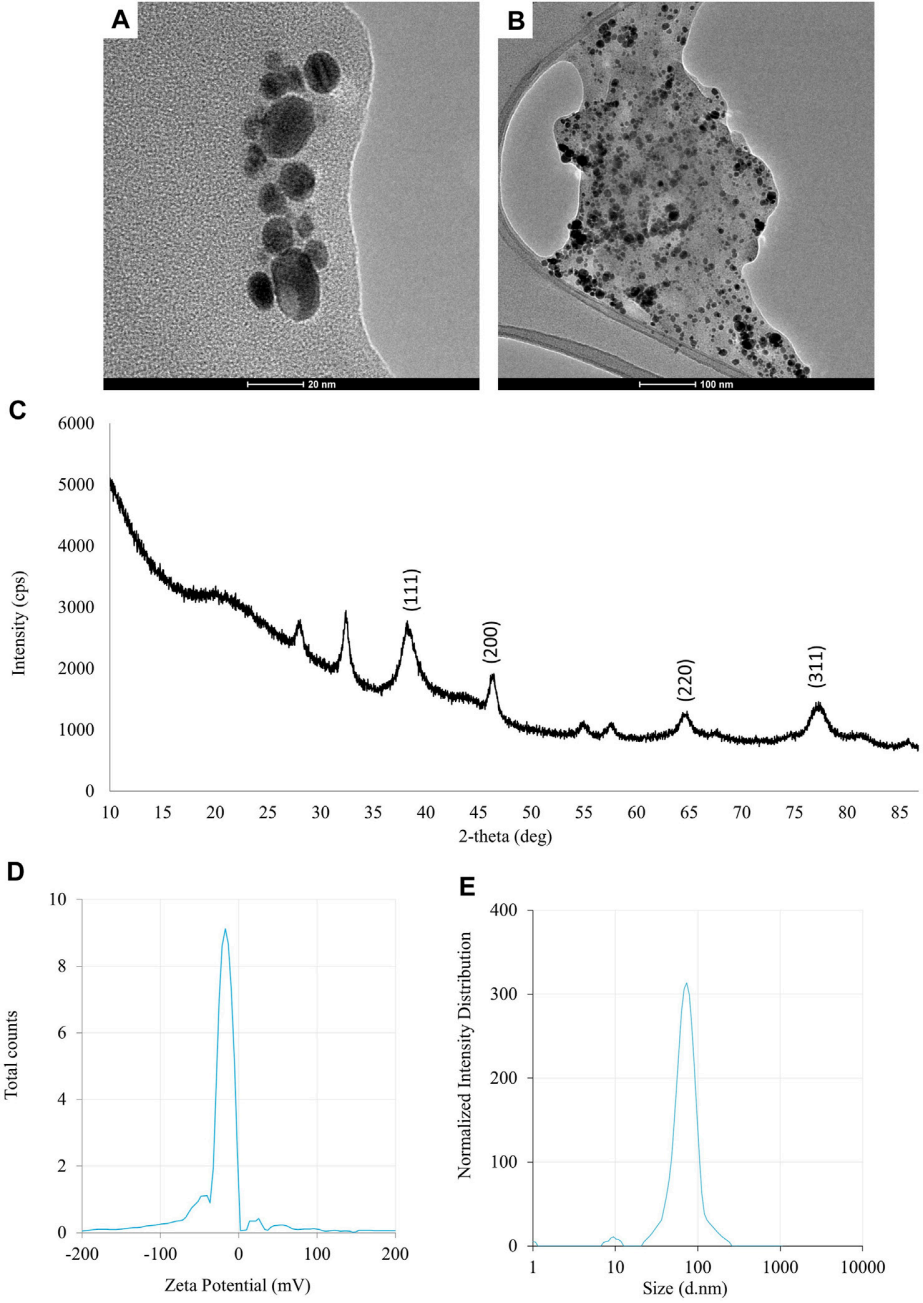
The results of the evaluation of physico-chemical properties of AgNPs synthesized from *Fusarium solani* IOR 825: TEM micrographs **(A, B)**, X-ray diffractogram **(C)**, Zeta potential **(D)** and particle diameter from DLS analysis **(E)**.

**FIGURE 2:**
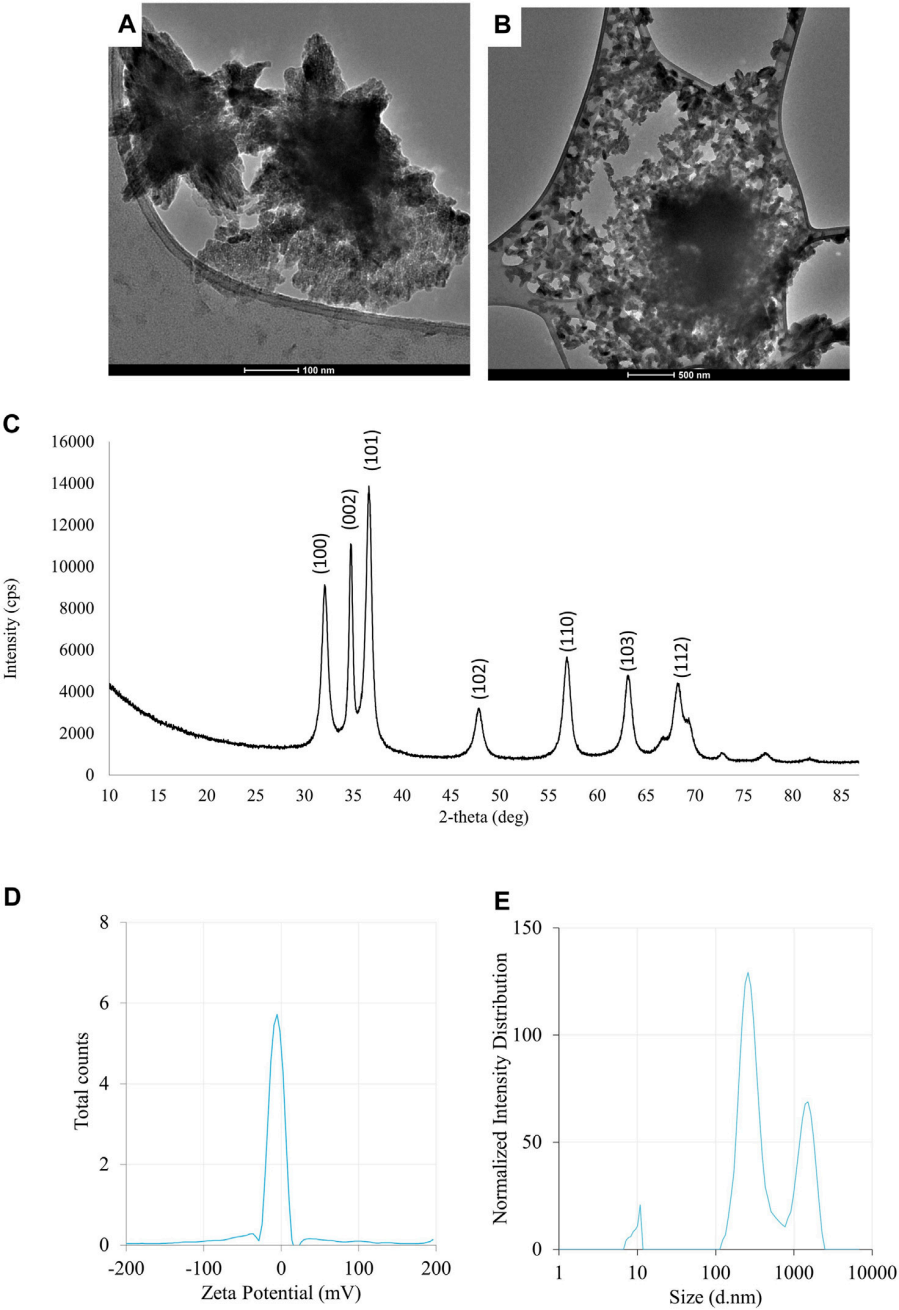
The results of the evaluation of physico-chemical properties of ZnONPs (1) synthesized from *Fusarium solani* IOR 825: TEM micrographs **(A,B)**, X-ray diffractogram **(C)**, Zeta potential **(D)** and particle diameter from DLS analysis **(E)**. (1); first method of ZnONP synthesis.

The UV-visible spectrometry of fungal extract from *F. solani* IOR 825 combined with AgNO_3_ showed a maximum absorbance peak at 420 nm and indicated the formation of AgNPs (Supplementary Figure S1 **)**. Synthesis efficiency was estimated at 26.35 mg of AgNPs per 100 mL of fungal extract as presented in Supplementary Table S1. Fungal-mediated synthesis resulted in the formation of spherical and oval-shaped small AgNPs with an average size of 8.27 ± 3.07 and sizes ranging from 2.99 to 21.53 nm (Figures 1A, B). The EDX studies of AgNPs displayed silver and carbon contents at 55.43% and 44.56%, respectively (Supplementary Table S2). X-ray pattern of AgNPs demonstrated peaks at 38.20, 46.31, 64.59, and 77.58 corresponding to reflections of the crystallographic planes (111), (200), (220), and (311), respectively, and revealed the formation of AgNPs (Figure 1C). AgNPs showed Zeta potential of −17.08 mV, while hydrodynamic diameter ranged from 20.6 to 260.5 nm with the highest frequency in the size of 68.3 ± 1.23 nm (Figures 1D, E). FTIR spectra of synthesized AgNPs showed adsorption bands at 3429.39, 2923.83, 2852.66, 1743.48, 1631.79, 1384.43, 1353.79, 1082.81 and 607.31 cm^-1^ (Figure 2).

**FIGURE 3:**
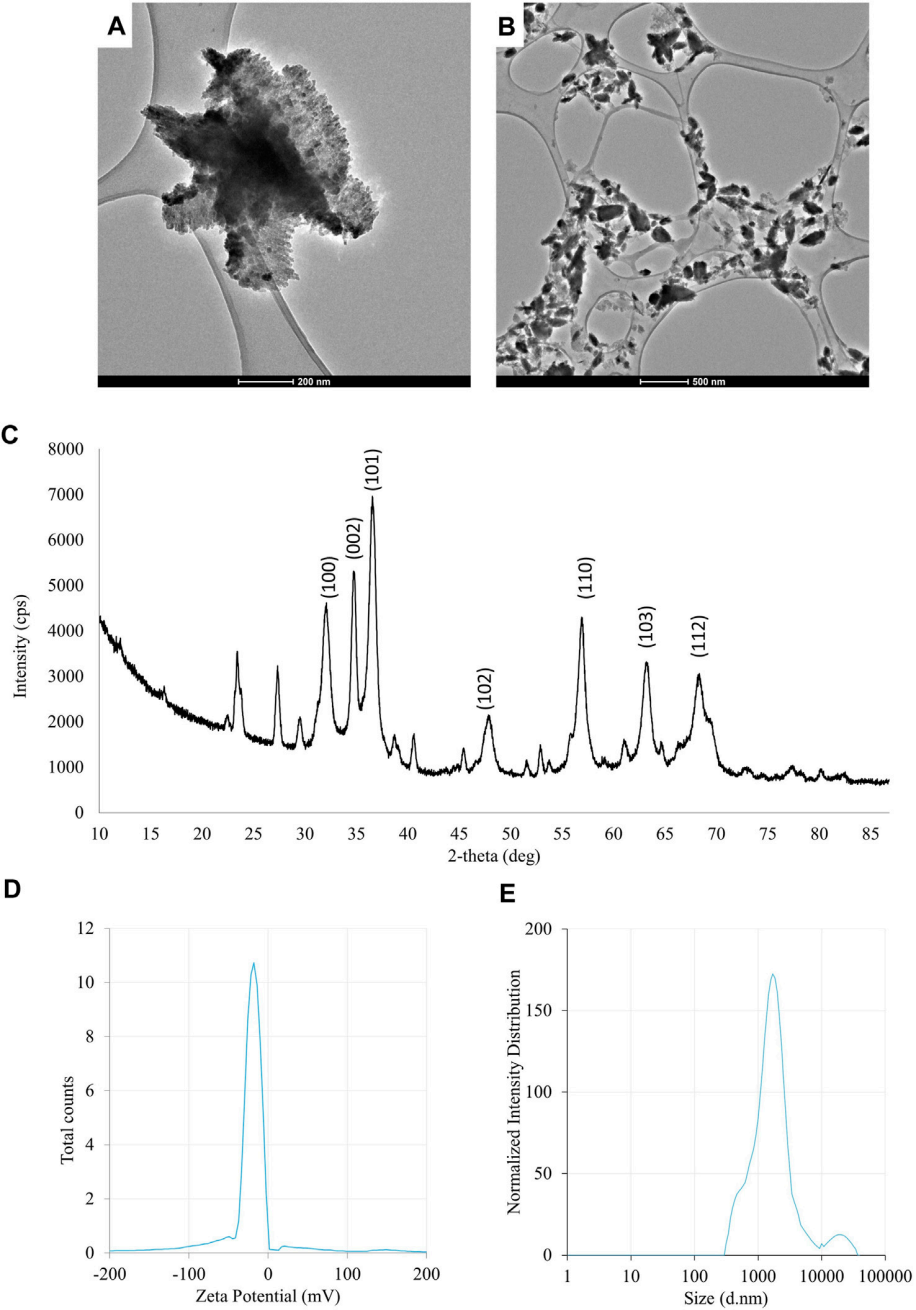
The results of the evaluation of physico-chemical properties of ZnONPs (2) synthesized from *Fusarium solani* IOR 825: TEM micrographs **(A,B)**, X-ray diffractogram **(C)**, Zeta potential **(D)** and particle diameter from DLS analysis **(E)**. (2); second method of ZnONP synthesis.

The synthesis of ZnONPs (1) was observed as a white precipitate in the reaction mixture and confirmed by the presence of maximum absorbance peak at wavelength 375 nm (Supplementary Figure S1). The synthesis yield of ZnONPs (1) was found to be 435.56 mg per 100 mL of fungal extract (Supplementary Table S1). TEM micrographs of ZnONPs (1) showed irregularly shaped structures with an average size of 117.79 ± 4.71 and a size ranging from 54.44 to 209.69 nm (Figures 3A, B). ZnONPs (1) consisted of 70.94% of zinc, 18.76% of oxygen and 10.03% of other minor elements (Mo, Al, Si) (Supplementary Table S2). The diffractogram of ZnONPs (1) showed peaks at 32.10, 34.80, 36.60, 48.10, 57.00, 63.00, 68.40 corresponding to (100), (002), (101), (102), (110), (103), (200), (112), (201) lattice plane values, respectively, and was recognized as hexagonal wurtzite phase of ZnO (Figure 3C). ZnONPs (1) were found to be negatively charged (−9.39 mV), with hydrodynamic diameters from 112.9 to 2495.9 nm and the largest fraction of size of 261.23 ± 53.5 (Figures 3D, E). The FTIR spectrum showed peaks at 3393.27, 2961.51, 2926.43, 1589.56, 1553.60, 1512.21, 1388.66, 1352.63, 1329.21, 1119.10, 1045.11, 940.98, 830.97, 738.83, 694.20, 608.67 and 480.24 cm^-1^ (Figure 2).

**FIGURE 4:**
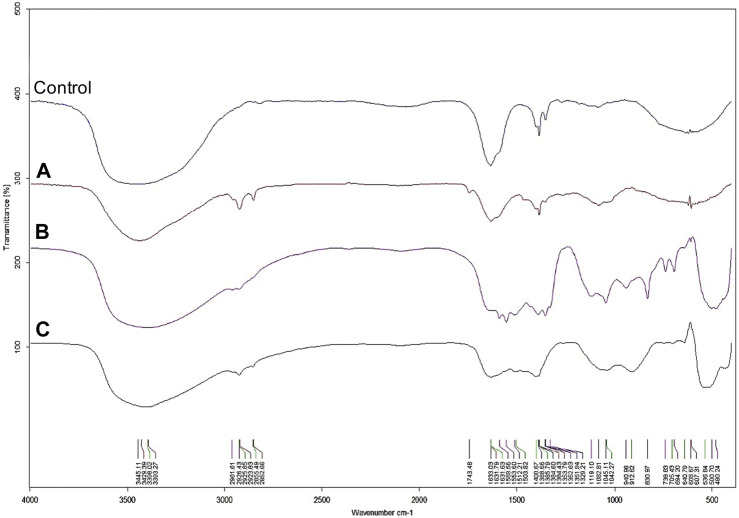
FTIR spectra of AgNPs **(A)**, ZnONPs (1) **(B)**, ZnONPs (2) **(C)** synthesized from *Fusarium solani* IOR 825. (1); first method of ZnONP synthesis, (2); second method of ZnONP synthesis.

The UV-vis spectrum of ZnONPs (2) demonstrated an adsorption peak at the wavelength of 375 nm (Supplementary Figure S1). The synthesis efficiency reached 525.8 mg of NPs per 100 mL of fungal extract (Supplementary Table S1). TEM analysis displayed nanorods-like NPs with an average length 175.12 ± 7.96 and size ranging from 64.84 to 443.02 nm (Figures 4A, B). The elemental composition from the EDX analysis demonstrated 78.67% of zinc, 19.35% of oxygen and 1.98% of carbon and aluminum (Supplementary Table S2). The XRD peaks at 32.10, 34.80, 36.60, 48.10, 57.00, 63.00, 66.00, 68.40, 69.55 were assigned to (100), (002), (101), (102), (110), (103), (200), (112), (201) lattice plane of hexagonal wurtzite phase of ZnO (Figure 4C). DLS analyses revealed NPs size between 292.9–9264.2 nm, with the maximum amount of NPs in the size of 1711.82 ± 123.59 nm while Zeta potential measurements showed negatively charged (−21.81 mV) ZnONPs (2) (Figures 4D, E). As shown in Figure 2, FTIR analysis revealed peaks at 3398.02, 2925.85, 2855.49, 1631.63, 1503.82, 1400.67, 1385.79, 1042.27, 912.62, 705.43, 640.79 and 536.84 cm ^-1^. Results from FTIR spectroscopy, summarized in Table 1, indicate the presence of various functional groups on the surface of NPs.

**TABLE 1:** An overview of functional groups identified on the surface of AgNPs and ZnONPs from *Fusarium solani* IOR 825 based on FTIR analysis.

Band position [cm^-1^], assigned functional groups	AgNPs	ZnONPs (1)	ZnONPs (2)
3393–3445 cm^−1^ (N–H stretching, primary amine)	+	+	+
2960–2925 (C-H stretching, aromatic compound)	+	+	+
2855 (O-H stretching, intramolecular bonded)	+	+	+
1740 (C-H bending, aromatic compound)	+	−	−
1632 (C=C stretching, alkene)	+	+	+
1590–1510 (C=O carbonyl group)	−	+	+
1450 (C-H bending, alkane, methyl group)	+	−	−
1400–1350 (C-H bending, aldehyde, alkane)	+	+	+
1120–1040 (C-N stretching, aromatic and aliphatic amine)	+	+	+
940–700 (C=C bending, alkene)	−	+	+
610–480 (metal)	+	+	+

+; − peak determined in an analyzed sample, −; peak not recorded in the analyzed sample.

ZnONP (1); first method of nanoparticle synthesis, ZnONP (2); second method of nanoparticle synthesis.

### 3.2 Antimicrobial activity

Antibacterial activity of NPs from *F. solani* IOR 825 against plant pathogens was determined based on minimal inhibitory (MIC) and biocidal (MBC) concentrations, as presented in Table 2.

**TABLE 2:** Antibacterial activity against phytopathogens of NPs synthesized from *Fusarium solani* IOR 825 presented as minimal inhibitory and biocidal concentrations (MICs and MBCs) [µg mL^-1^].

Bacterial phytopathogens	AgNPs		ZnONPs (1)		ZnONPs (2)	
	MIC	MBC	MIC	MBC	MIC	MBC
*Agrobacterium tumefaciens* IOR 911	128	1024	256	512	1024	1024
*Pectobacterium carotovorum* PCM 2056	256	1024	>2048	>2048	>2048	>2048
*Pseudomonas syringae* IOR 2188	8	512	1024	2048	512	>2048
*Xanthomonas camperstris* IOR 512	256	512	256	2048	512	>2048

ZnONP (1); first method of nanoparticle synthesis, ZnONP (2); second method of nanoparticle synthesis.

The highest antibacterial activity of AgNPs was observed against *Pseudomonas syringae,* followed by *Xanthomonas campestris, Agrobacterium tumefaciens* and *Pectobacterium carotovorum*; the MIC and MBC values ranged from 8 to 256 μg mL^-1^ and from 512 to 1024 μg mL^-1^, respectively.

The MIC values for ZnONPs (1) were determined at a concentration of 256 μg mL^-1^ against *A. tumefaciens* and *X. campestris*, and 1024 μg mL^-1^ against *P. syringae*. In turn, the corresponding MBC values of these nanoparticles were found to be 512, 2048 and 2048 μg mL^-1^, respectively.

The ZnONPs (2) showed inhibitory effect against *P. syringae* and *X. campestris* at a concentration of 512 μg mL^-1^ while the corresponding biocidal activity of NPs was not determined at the tested concentration range. The inhibitory and biocidal activities against *A. tumefaciens* were recorded at a concentration of 1024 μg mL^-1^.

None of the ZnONPs were found to be active against *P. carotovorum* at the tested concentration range.

The results of antifungal activity of AgNPs and ZnONPs are shown in Table 3. The food poisoned method revealed that *S. sclerotiorum* was most susceptible to AgNPs, followed by *Phoma lingam, Botrytis cinerea,* and both *Alternaria alternata* strains. The remaining strains were found to be less or not susceptible to AgNPs at the tested concentration range. Determination of MIC and MFC of AgNPs against fungal spores showed their highest activity against *S. sclerotiorum* (MIC and MBC values at 16 μg mL^-1^). The slightly lower antifungal activity (MIC and MBC = 32 μg mL^-1^) of AgNPs was noted for both *A. alternata* strains, *B. cinerea, Fusarium graminearum* D, *Fusarium oxysporum* D, *Fusarium poae* A and three *Phytophthora* strains. The AgNPs inhibited spore germination of *Aspergillus niger* at a concentration of 32 μg mL^-1^ while their biocidal effect was achieved at a concentration of 64 μg mL^-1^. Similarly, MIC and MFC of AgNPs against *P. lingam* spores were noted at 64 μg mL^-1^. The higher MIC and MBC values of AgNPs, from 64 to 2048 μg mL^-1^, were determined against the other tested microorganisms, with the exception of *F. oxysporum* IOR 342 for which the MBC values were not determined in the tested concentration range.

**TABLE 3:** Antifungal activity of NPs synthesized from *Fusarium solani* IOR 825 against phytopathogens presented as minimal inhibitory and minimal fungicidal concentrations (MICs and MFCs) [µg mL^-1^] and mycelial growth inhibition (MGI) [%].

	AgNPs				ZnONPs (1)				ZnONPs (2)			
	% MGI		MIC	MBC	% MGI		MIC	MBC	% MGI		MIC	MBC
NPs contentrations [µg mL^-1^]	100	200			100	1000			100	1000		
*Alternaria alternata*	-	57	32	32	-	52	1024	>2048	-	57	1024	>2048
*Alternaria alternata* IOR 1783	-	48	32	32	-	52	512	512	-	57	256	512
*Aspergillus niger*	-	-	32	64	-	19	512	2048	-	35	512	2048
*Botrytis cinerea* IOR 1873	45	58	32	32	-	-	1024	2048	-	-	1024	1024
*Colletotrichum acutatum* IOR 2153	-	-	64	64	-	-	1024	2048	-	-	1024	2048
*Fusarium culmorum*	-	-	64	2048	-	-	2048	2048	-	-	2048	2048
*Fusarium culmorum* IOR 2333	-	-	64	256	-	-	2048	2048	-	-	1024	1024
*Fusarium culmorum* DMS 114849	-	19	64	2048	-	25	2048	2048	-	23	2048	2048
*Fusarium graminearum* A	-	-	64	256		47	2048	2048	-	53	1024	1024
*Fusarium graminearum* D	-	-	32	32	-	-	2048	2048	-	-	1024	>2048
*Fusarium oxysporum*	-	-	64	64	-	-	2048	2048	-	-	1024	2048
*Fusarium oxysporum* IOR 342	-	-	128	>2048	40	100	128	512	62	100	256	256
*Fusarium oxysporum* D	14	20	32	32	-	31	2048	2048	22	34	2048	2048
*Fusarium poae* A	-	-	32	32	-	38	1024	2048	-	37	2048	2048
*Fusarium tricinctum*	-	-	64	64	-	-	2048	2048	-	-	>2048	>2048
*Penicillium* sp.	-	-	64	1024	-	-	2048	>2048	-	-	2048	>2048
*Penicillum spinulosum*	-	-	64	1024	-	-	2048	>2048	-	-	512	>2048
*Phoma lingam* IOR 2284	49	61	64	64	39	63	2048	1024	29	60	1024	1024
*Sclerotinia sclerotiorum* IOR 2242	52	100	16	16	-	72	512	512	-	72	1024	1024
*Phytophthora megasperma* IOR 404	-	-	32	32	-	-	>2048	>2048	-	-	>2048	>2048
*Phytophthora cryptogea* IOR 2080	-	-	32	32	-	-	>2048	>2048	-	-	>2048	>2048
*Phytophthora plurivora* IOR 2303	-	-	32	32	-	-	>2048	>2048	-	-	>2048	>2048

ZnONP (1); first method of nanoparticle synthesis, ZnONP (2); second method of nanoparticle synthesis.

(−); no antifungal activity.

The highest mycelial growth inhibition, determined by food poisoned technique, was found for *F. oxysporum* IOR 342, followed by *S. sclerotiorum, P. lingam* and both *A. alternata* strains, when treated with ZnONPs (1). MIC and MFC of ZnONPs (1) against fungal spores were found in the range of 128–2048 μg mL^-1^. The most sensitive to ZnONPs (1) were spores of *F. oxysporum* IOR 342; the inhibitory and biocidal effects were observed at a concentration of 128 and 512 μg mL^-1^, respectively. The high susceptibility to ZnONPs (1) was also observed for *A. alternata* IOR 1783 and *S. sclerotiorum* IOR 2242 (MIC and MBC of 512 μg mL^-1^).

In the case of ZnONPs (2) similar pattern was observed, as described for ZnONPs (1), with exception for MIC and MBC values against *S. sclerotiorum* which were found to be two times higher (Table 3).

None of ZnONPs showed spore inhibition activity, within the concentration ranges tested, against oomycete strains from the genus *Phytophthora* (Table 3).

### 3.3 Effect of NPs on germination and growth of maize (*Zea* mays) seedlings

The pretreatment of maize seeds with AgNPs at concentrations equal to or higher than 32 μg mL ^-1^ was found to be an effective method of seed surface sterilization. However, priming of seeds with ZnONPs within tested concentration range (1–256 μg mL ^-1^) showed no sterilization effect. The sterilization of seeds with AgNPs and seeds priming with ZnONPs had no significant effect on maize seed germination, when compared to the control (Table 4).

**TABLE 4:** Germination parameters of maize seeds after sterilization with various concentrations of AgNPs and pretreatment with ZnONPs (1) and ZnONPs (2).

	Treatment [µg mL^-1^]	% germination	MGT [day]	GRI [%/day]	Vigour index I	Vigour index II
AgNPs	0	90.00	3.78	28.01	2206.16	3957.62
	32	82.50	3.61	29.14	2304.39	4326.20
	64	82.50	3.64	28.89	2436.06	4363.58
	128	87.50	3.66	29.00	2427.12	4347.94
	256	87.50	3.57	29.52	2335.58	3884.63
ZnONPs (1)	0	90	3.78	28.01	2209.35	3957.62
	1	92.5	3.78	27.93	2240.51	3806.82
	8	97.5	3.64	29.19	2324.32	4168.38
	16	90	3.86	27.18	2584.22	4711.75
	32	92.5	3.81	27.48	2854.74	5316.10
	64	90	3.69	28.52	2754.78	4910.63
	128	90	3.75	28.15	2803.71	4932.74
	256	95	3.72	28.38	3036.71	5247.18
ZnONPs (2)	0	90	3.78	28.01	2209.35	3957.62
	1	90	3.76	28.07	2416.50	4234.28
	8	90	3.53	29.95	2498.03	3994.16
	16	100	3.83	27.38	2996.54	5199.35
	32	90	3.83	27.31	2653.71	4602.11
	64	90	3.69	28.52	2792.48	4710.99
	128	92.5	3.73	28.29	3038.07	5111.09
	256	95	3.74	28.20	3093.61	5150.36

ZnONP (1); first method of nanoparticle synthesis, ZnONP (2); second method of nanoparticle synthesis.

MGT; mean germination time, GRI; germination rate index.

**FIGURE 5:**
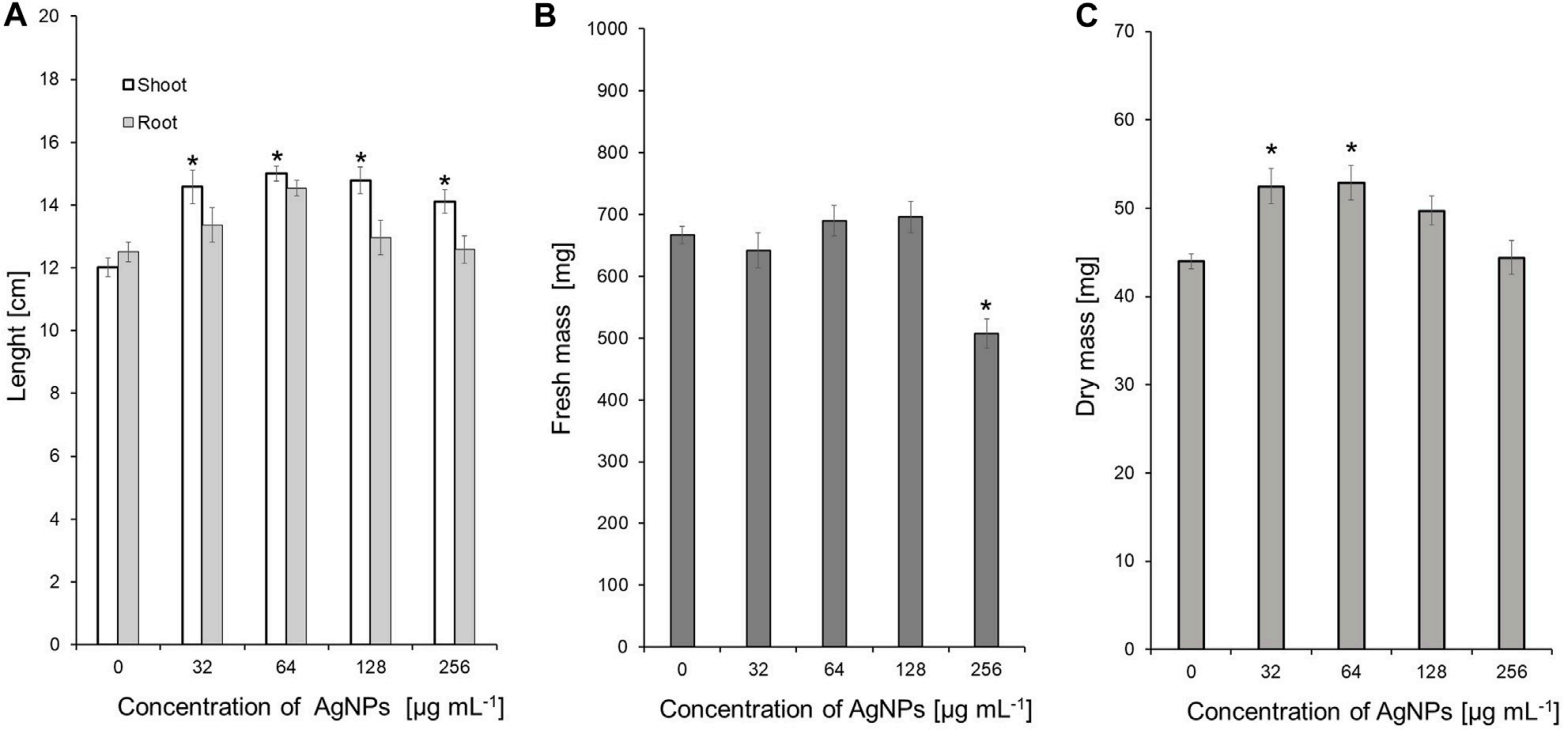
The length of shoots and roots **(A)**, as well as fresh **(B)** and dry weight **(C)** of 10-day-old maize seedlings after sterilization of seeds with AgNPs. Data presented as mean and standard error (SE), * denote statistical significance (*p*-value <0.05) between AgNPs treatment and control.

Values of both vigor indexes (I and II) (Table 4) showed an acceleration of seedling growth after treatment with AgNPs at concentrations of 32, 64 and 128 μg ml ^-1^, as indicated by significantly longer shoots and higher seedling dry weight (Figures 5A, C). However, application of the maximum tested concentration of AgNPs (256 μg ml ^-1^) resulted in significantly lower fresh biomass of seedlings (507.11 mg) when compared to control (666.85 mg) (Figure 5B). Although AgNPs were used for sterilization purpose, their lowest effective concentrations (32 and 64 μg mL^-1^) significantly improved parameters of seedlings, especially seedling length (13.9%–20.5%) and dry mass (19.3%–20.3%), as summarized in Table 5.

**TABLE 5:** Variation in the growth parameters of maize seedlings after treatment of seeds with various concentrations of NPs compared to the control expressed as %.

Treatment [µg mL^-1^]	Seedling length			Fresh mass			Dry mass		
	AgNPs	ZnONPs (1)	ZnONPs (2)	AgNPs	ZnONPs (1)	ZnONPs (2)	AgNPs	ZnONPs (1)	ZnONPs (2)
1		−1.3	9.4		−6.1	−1.4		−6.4	7.0
8		−2.9	13.1*		−4.9	−5.2		−2.8	0.9
16		17.0	22.1*		24.2*	23.8*		19.1*	18.2*
32	13.9*	25.7*	20.1*	−3.7	32.1*	24.9*	19.3*	30.7*	16.3*
64	20.5*	24.7*	26.4*	3.5	25.7*	27.6*	20.3*	24.1*	19.0*
128	13.2*	26.9*	33.8*	4.3	29.5*	40.5*	13.0	24.6*	25.7*
256	8.9	30.2*	32.7*	−24.0*	30.0*	34.0*	1.0	25.6*	23.3*

*Denote statistical significance (*p*-value <0.05) between NPs, treatment and control.

ZnONP (1); first method of nanoparticle synthesis, ZnONP (2); second method of nanoparticle synthesis.

**FIGURE 6:**
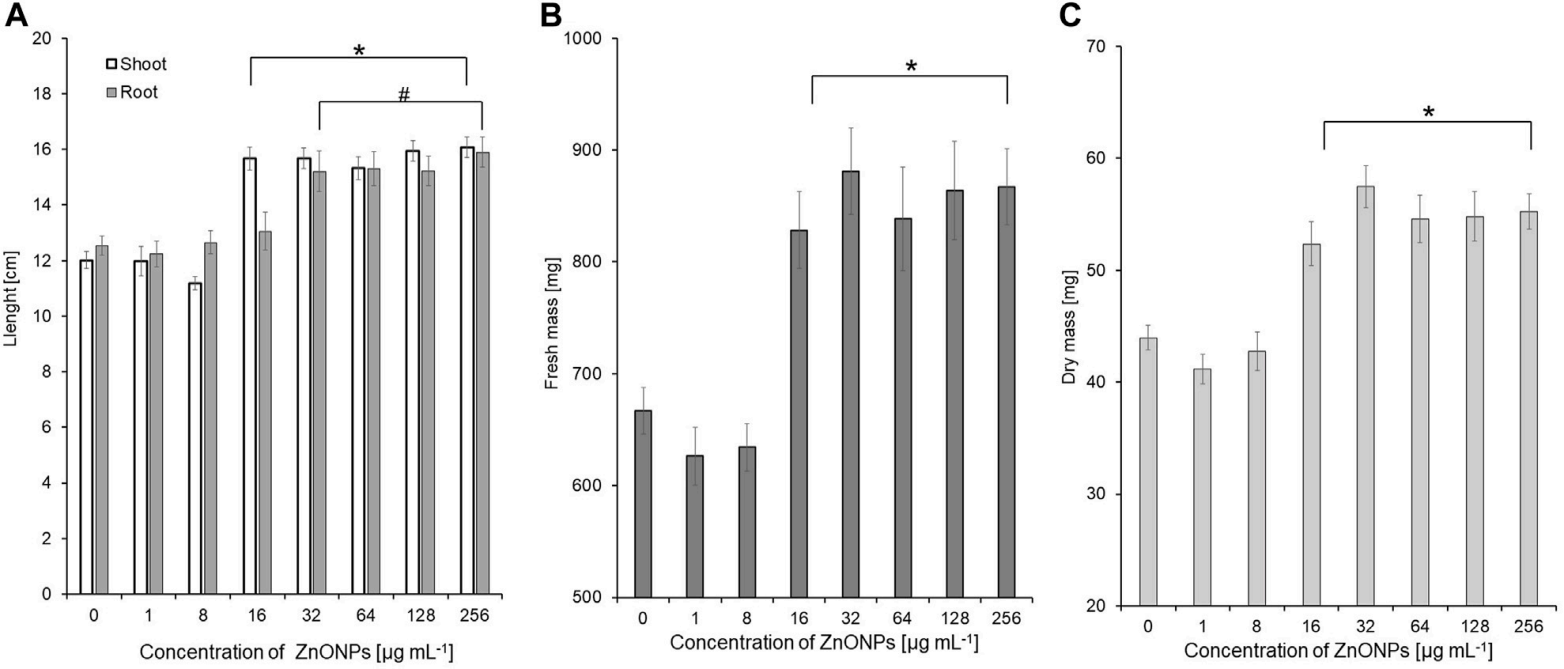
The length of shoots and roots **(A)**, as well as fresh **(B)** and dry weight **(C)** of 10-day-old maize seedlings after seed pretreatment with ZnONPs (1) at different concentrations. Data presented as mean and standard error (SE), * and # denote statistical significance (*p*-value <0.05) between ZnONPs treatment and control. (1); first method of ZnONP synthesis

The vigour indexes of seedlings were found to be higher after treatment with ZnONPs (1) at concentrations above 16 μg ml ^-1^. Seedling vigour index I increased after treatment with ZnONPs (1) at concentrations of 16–256 μg ml ^-1^ from 2209 in control to 2584–3037 in tested samples. The highest vigour index II (5316) was found at concentrations of 32 μg ml ^-1^ **(** Table 4). Growth of seedlings roots and shoots was improved by ZnONPs (1) at concentrations of ≥16 and ≥32 μg ml ^-1^, respectively (Figures 6A, B). ZnONPs (1) stimulated the growth of maize seedlings as indicated by higher plant fresh and dry weight. A statistically significant increase in maize biomass production was noted for ZnONPs (1) at concentrations of ≥16 μg ml ^-1^, when compared to the control (Figures 6C, D). Overall, the growth parameters of maize seedlings were increased after seeds pretreatment with ZnONPs (1) at concentration ranges between 32 and 256 μg mL^-1^, namely, length (by 26%–30%), fresh weight (by 24%–30%), dry weight (by 19%–30%) (Table 5).

**FIGURE 7:**
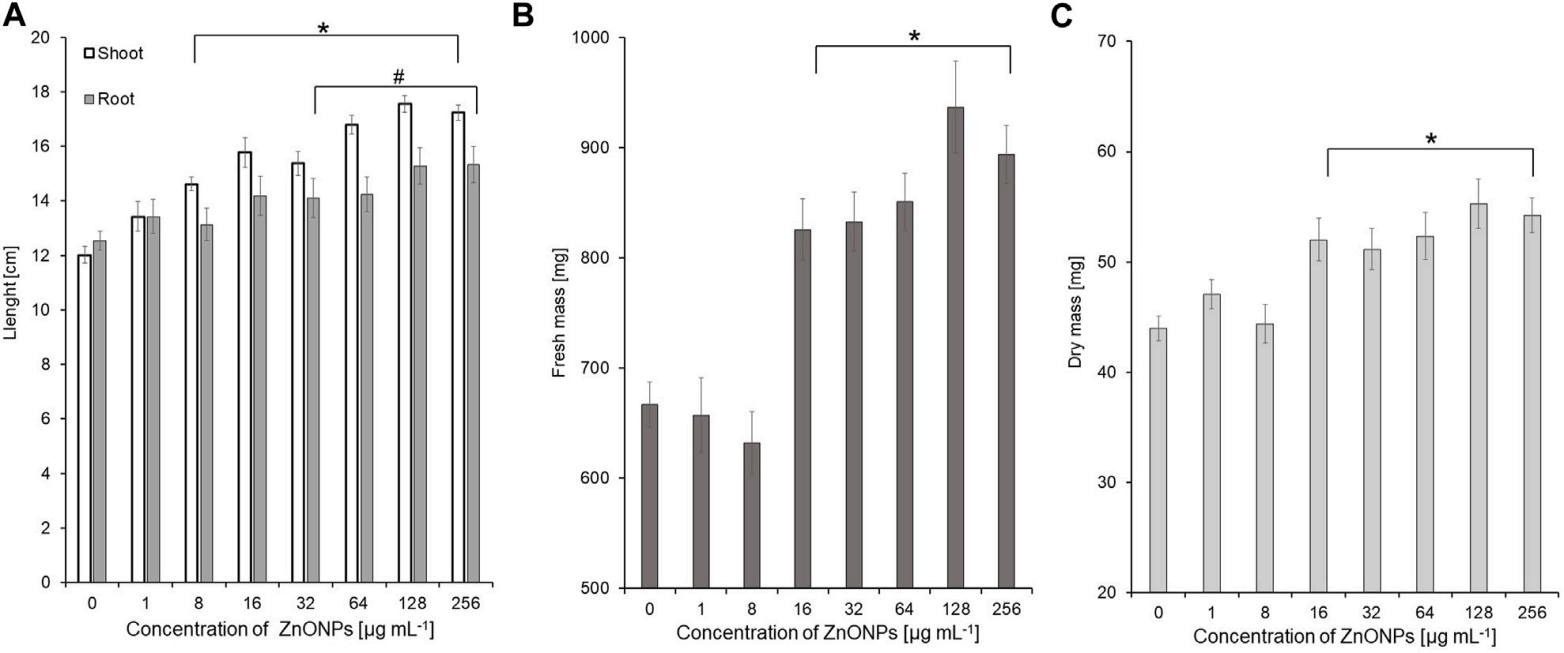
The length of shoots and roots **(A)**, as well as fresh **(B)** and dry weight **(C)** of 10-day-old maize seedlings after seed pretreatment with ZnONPs (2) at different concentrations. Data presented as mean and standard error (SE), * and # denote statistical significance (*p*-value <0.05) between ZnONPs treatment and control. (2); second method of ZnONP synthesis.

Seedling vigour index I after treatment with ZnONPs (2) at concentrations of 8–256 μg ml ^-1^ increased to 2498–3094 in tested seedlings when compared to control (2209). The strongest effect on improving seedling condition was observed after treatment of maize seeds with ZnONPs (2) at a concentration of 16 μg ml ^-1^, as proved by seedling vigour indexes (5199) when compared to untreated control (3958) (Table 4). ZnONPs (2) concentrations ≥8 and 128 μg ml ^-1^ stimulated elongation of shoots and roots, respectively (Figure 7A). The fresh and dry weight of seedlings increased after seeds priming with ZnONPs (2); a statistically significant difference was noted for concentrations of ≥16 μg ml ^-1^, when compared to the control (Figures 7B, C). To sum up, parameters of maize seedlings were increased after seed pretreatment with ZnONPs (2) at concentration ranges between 32 and 256 μg mL^-1^. The length of seedlings was improved by 13%–34%, their fresh weight by 24%–40% and dry weight by 16%–26%, as presented in Table 5.

## 4 Discussion

Fungi play a pivotal role in synthesis of different kinds of nanoparticles. Among these, various *Fusarium* species have garnered attention by the researchers (Rai et al., 2021). Several researchers successfully synthesized variety of nanoparticles including silver, gold, copper, and zinc oxide NPs synthesis from different strains of *F. solani* (Ingle et al., 2009; Abd El-Aziz et al., 2015; Vijayan et al., 2016; Clarance et al., 2020; El Sayed and El-Sayed, 2020; Sasani et al., 2023). Nevertheless, there is no standard synthesis protocol, as there are many factors that affect the synthesis yields of final product sizes, shapes, and other physicochemical characteristics (Sonawane et al., 2022). Therefore, the present work focuses on optimization of *F. solani* IOR 825 growth, high-throughput synthesis process, and fabrication of biologically active nanoparticles. The main requirements for the biosynthesis of NPs are solutions of metal ions as well as reducing and coating agents (fungal origin), the initial aim was to optimize the growth of the fungus in order to acquire a large portion of biomass, with a view to its subsequent use in preparing a cell-free extract after autolysis. The SDB medium was selected and used for further culture of *F. solani* IOR 825, as the highest fungal growth rate was recorded when compared with PDB or CDB, which may be due to the medium content consisting of dextrose, digested animal tissues (amino acids source) and casein, which provide carbon and nitrogen sources for growth. Similarly, Merlin et al. (2013) pointed out dextrose as the preferred carbon source, and the supplementation of amino acids to the growth medium that resulted in increased growth of *F. solani* strain LCPANF01.

The fungal extract obtained after cell autolysis, which contains variable biomolecules, is preferably used for the synthesis of NPs along with metal precursors. This extracellular process allows for more efficient synthesis of NPs and reduction of post-production steps, such as purification (Michael et al., 2022). Similarly, El Sayed and El-Sayed (2020) used cell-free extracts from *F. solani* KJ 623702 and silver nitrate (AgNO_3_; final concentration of 0.5 mM) or zinc sulfate (ZnSO_4_; 0.005 mM), as precursors, for green synthesis of corresponding nanoparticles. A basic visual observation of changes in the color of the mixture to dark brown (AgNPs) or white (ZnONPs) indicated that the synthesis reaction had been initiated. The reduction of metal ions and nanoparticles formation were confirmed by UV-visible spectroscopy and the detection of maximum absorbance peaks at wavelengths 422 nm and 375 nm for AgNPs and ZnONPs, respectively (El Sayed and El-Sayed, 2020), which is in line with our observations. It is well known, that the maximum absorption for AgNPs and ZnONPs ranges from 420 to 450 nm and 330–380 nm, respectively (Musa et al., 2017; Anjum et al., 2023). However, the slight shifts in the UV-vis spectra are due to differences in particle size or the presence of molecules from the biological extracts used for the synthesis (Saion et al., 2013; Ballotin et al., 2016; Musa et al., 2017; Guilger-Casagrande et al., 2019; Urnukhsaikhan et al., 2021). A number of studies on the synthesis of ZnONPs by precipitation method using fungal extracts have been reported (Ghorbani et al., 2015). In our study, two factors, namely, temperature, and pH, were considered for ZnONPs synthesis, as these factors are of great importance for the morphology and size of formed NPs (Moezzi et al., 2011). The heating of the reaction mixture (fungal extract and salt precursor) improves the kinetics of the reaction and affect NPs properties (Moezzi et al., 2011). Authors reported that an increase in the temperature of the reaction from 25°C to 90 °C between addition of ZnSO_4_ and NaOH led to the lower solubility of the final product, which is important for further applications. In another study, Abdullah et al. (2020) indicated that strong alkaline (pH > 10) reaction environment favors the formation of ZnONPs. It is consistent with observation from this study, the ZnONPs were formed after a temperature rise to 40°C or at room temperature after adjusting the pH to 11.

TEM analysis showed the formation of small and spherical AgNPs from extract of *F. solani* IOR 835 which is in line with recently published reports on the fungal synthesis of such nanoparticles (Lofty et al., 2021; Sasani et al., 2023). These authors synthesized spherical and small (7–23 and 27.5–58.3 nm) AgNPs from *Aspergillus terreus* and *F. solani*, respectively. In contrast, ZnONPs synthesized from *F. solani* IOR 825 were irregularly shaped and bigger (54.44–209.69 nm and 64.84–443.02 nm) than those synthesized from *Trichoderma asperellum* (44–78 nm), it may be related to the use of another salt precursor (Zn (NO_3_)_2_ (Shobha et al., 2023). Whereas, Elrefaey et al., 2022 synthesized rectangular ZnONPs with size between 23 and 140 nm using extract of marine alga *Cystoseira crinite,* Zn SO_4_ (0.05 M aqueous solution), as a precursor, and 1 M NaOH and heating the reaction mixture up to 45°C for 30 min. Apart from the reaction parameters (precursor, temperature, pH), the physicochemical properties of the bio-NPs are determined by the composition of the fungal extract (Lofty et al., 2021). Similar results, confirming the fungal-mediated formation of AgNPs and ZnONPs with crystalline structures were reported by other researchers (Talam et al., 2012; Nallal et al., 2021; Shobha et al., 2023). Furthermore, Singh D. et al. (2014) revealed that low concentration of precursor (1 mM AgNO_3_) as well as room temperature (below 25°C) allowed for efficient mycosynthesis of small AgNPs (average size 18 nm) with good stability (Zeta potential–33.4 mV) which prevented their agglomeration. Jain et al. (2020) used zinc sulfate for microbial-mediated synthesis of ZnONPs and suggested that a larger hydrodynamic diameter of biologically synthesized ZnONPs might result from aggregation of particles. The zeta potential is an indicator of surface charge potential which is an important parameter for understanding the stability of nanoparticles in aqueous suspensions. In our studies, a low value of Zeta potential (−9.39 mV) indicated lower stability of ZnONPs (2) and the tendency to aggregate therefore larger particles diameter were observed in DLS analysis. It has been stated in the literature that nanoparticles with a Zeta potential higher than +30 mV or lower than −30 mV are considered to be very stable in the dispersion medium by pushing the same charges (Rai et al., 2018). In another study, Singh K. et al. (2014) pointed out that the DLS results encompass the layer of solvent at the interface and capping biomolecules on the nanoparticles’ surface. This was also indicated by the results of FTIR analysis, where bands identified suggest the presence of functional groups from biomolecules such as proteins (N–C-and C–C), aromatic compounds (C-H), amines (C-N), hydrocarbons (C-H, C=C) from the fungal extract which are employed in the synthesis and take part in the reduction of metal ions and subsequent stabilization of the nanoparticles (Abdullah et al., 2020; Wypij et al., 2022). The bands detected between 400 cm ^-1^–600 cm ^-1^ imply the formation of bonding between metal and biomolecules present in fungal extract. Our results agree with the findings reported by Salem (2022) and Shobha et al. (2023) where bands for biosynthesized nanoparticles were found at 609.3 and 414.6 cm^−1^ for AgNPs and 534 cm^−1^ for ZnONPs, respectively.

Mycosynthesized NPs from *F. solani* IOR 825 showed antimicrobial activity against a wide range of bacterial and fungal pathogens of plants in a dose-dependent manner, highly depending on the targeted strain. In a similar study by Namburi and coworkers (2021), antibacterial activity of biosynthesized AgNPs was found against *Xanthomonas oryzae* pv. *oryzae* at concentration of 2.5 μg mL^-1^ while another report indicated effectiveness of AgNPs synthesized from *Ulva fasciata* extract against *X. campestris* pv. *malvacearum* at higher concentration of 40 μg mL^-1^ (Rajesh et al., 2012). In a study conducted by Manosalva et al. (2019) AgNPs showed activity against *E. coli*, *P. syringae,* and *Staphylococcus aureus*. The activity of AgNPs was affected by both the bacterial strain and the size of the NPs (23, 92, and 220 nm); the highest sensitivity to AgNPs was detected for *Escherichia coli* (MIC 5–30 μg mL^-1^ and MBC 10–50 μg mL^-1^)*,* followed by *P. syringae* (MIC 10–40 μg mL^-1^ and MBC 30–60 μg mL^-1^) and *S. aureus* (MIC 50–80 μg mL^-1^ and MBC 60–80 μg mL^-1^). In addition, the growth of bacterial strains, namely, *Ralstonia solanacearum*, *P. syringae*, *X. campestris,* and *X. oryzae* was inhibited by ZnONPs synthesized from *C. tomentosa* leaf extract at various concentrations of 125, 500, 250 and 250 μg mL^-1^, respectively. A similar trend was found in our study, where *P. syringae* showed lower sensitivity to ZnONPs than *X. campestris.* The growth of *P. syringae* was inhibited by ZnONPs (1) and ZnONPs (2) at concentrations of 1024 and 512 μg mL^-1^, respectively, while *X. campestris* at concentrations of 256 and 512 μg mL^-1^, respectively. Several studies indicated that antibacterial mechanisms of metal nanoparticles include the destruction of membrane integrity, cell morphology changes, the release of metal ions, and the generation of reactive oxygen species generation (Reddy et al., 2007; Jiang et al., 2016; Gallón et al., 2019). To date, the antifungal activity of biosynthesized AgNPs and ZnONPs were tested against plant pathogens, e.g., *Alternaria brassicae* (Dhiman et al., 2021), *F. oxysporum* (González-Merino et al., 2021; Macías Sánchez et al., 2023) and *F. graminearum* (Ibrahim et al., 2020). For example, Talie et al. (2020) showed lower antifungal activity of AgNPs biosynthesized from *Helvella leucopus* which at a concentration of 20 mg mL^-1^ inhibited spore germination of *Penicillium chrysogenum, A. niger,* and *A. alternata* by 83.21, 77.32% and 69.10%*,* respectively. The dose-dependent antifungal activity of AgNPs biosynthesized from *Trichoderma viride* against *F. oxysporum* and *Alternaria brassicicola* was reported by Kumari et al. (2019). These results corroborate our studies. Inhibition of mycelial growth was observed at AgNPs concentration of 5%, while complete suppression was determined at a nanoparticle concentration of 25%. In addition, they suggested that the action of AgNPs against *A. brassicicola* led to the generation of superoxide radicals, as well as the disruption of the mycelial structure (Kumari et al., 2019). Jain et al. (2020) evaluated antimicrobial activity of bio-ZnONPs against *X. oryzae* and *Alternaria* sp. with the maximum effect at concentration of 100 and 250 μg ml^−1^, respectively. Similary to the results of the present work, Jamdagni et al. (2018) found that ZnONPs synthesized from *Nyctanthes arbor-tristis* flower extract showed MIC values of 16 μg mL^-1^ against *A. niger*, 64 μg mL^-1^ against *A. alternata* and *F. oxysporum*, and 128 μg mL^-1^ against *B. cinerea* and *Penicillium expansum*. Whereas, Zhu et al. (2021) reported inhibitory effect of ZnONPs synthesized from *Cinnamomum camphora* (L.) leaf extract on mycelial growth and spore germination of *A. alternata* at concentrations of 20–160 mg mL^-1^. The proposed mechanisms of antifungal activity of ZnONPs included excessive synthesis and accumulation of malondialdehyde in *A. alternata* cells and damage to the cell membrane, leading to leakage of proteins and nucleic acids (Zhu et al., 2021). In turn, Nandhini et al. (2019) observed the plasmolysis of spores of *Sclerospora graminicola* after treatment with ZnONPs at a concentration of 50 ppm. The differences in antimicrobial activity of both types of ZnONPs biosynthesized from *F. solani* IOR 825 may result from different protocols used for their biofabrication. There are several important factors that affect the synthesis of nanoparticles, including pH of the reaction solution, temperature, pressure and time of the reaction, the concentration of the extracts and precursors, and above all the protocols that are followed for the synthesis process (Patra and Baek, 2014; Wypij et al., 2019). Consequently, nanoparticles of different sizes, shapes, structures and properties are formed which affect their biological activity, including antimicrobial activity (Patra and Baek, 2014; Wypij et al., 2019), as discussed above.

A notable antimicrobial activity of mycosynthesized AgNPs was further confirmed in seeds sterilization tests. The minimum concentration of AgNPs that effectively sterilized maize seeds (32 μg mL^-1^) was equal to or twice lower than the majority of MICs determined against the tested bacterial and fungal plant pathogens. It is noteworthy that at this concentration no negative effects of AgNPs on seed germination or seedling growth were observed, as mentioned previously. Previously, Morsy et al. (2014) used bioAgNPs form cyanobacteria for sterilization of maize, sorghum, soybean and sesame seeds. Although the authors used 2.5 higher AgNPs dose than in the present study, they noted incomplete sterilization of seeds indicated by the presence of fungi from genera *Fusarium* and *Alternaria* sp. *Aspergillus* spp. or *Pencillium* spp. The results of the present study showed that overall AgNPs at tested concentration range did not negatively affected seedling parameters, except of fresh biomass production at concentration of 256 μg mL^-1^. It may suggest phytotoxicity of AgNPs at higher doses. Similarly, dose-dependent plant responses to AgNPs priming of seeds were reported by other authors. In the study by Labeeb et al. (2020), AgNPs at concentrations of 20 and 40 mg L^-1^ increased germination of seeds and root length of green pea (*Pisum sativum* L.), while higher concentrations (80 and 160 mg L^-1^) decreased seed germination and reduced seedling growth. It has been suggested that the phytotoxicity of AgNPs, especially at higher doses, may be related to their small size which facilitates their transport. Once penetrating into plant tissues and cells, they display cytotoxic and genotoxic effects (Scherer et al., 2019).

Although ZnONPs from *F. solani* IOR 825 were found to display non-sterilizing properties, they significantly improved seedling growth by stimulating shoot and root elongation, and fresh and dry matter production. A number of reports have proven the importance of Zn for plant growth and development as well as their resistance to biotic or abiotic stresses, that result from its involvement in physiological processes (Camp, 1945; Rudani et al., 2018). Zn content is essential for plants as a component of the cytochrome complex, for membrane integrity or as a cofactor or complexing ion for enzyme activity (Hacisalihoglu, 2020). Some other reports highlight a significant role of Zn in cell elongation and synthesis of tryptophan, a precursor of indole-3-acetic acid (Mašev and Kutáček, 1966; Sharifi et al., 2016; Sharma et al., 2021). The beneficial effects of ZnONPs on seeds germination and early seedling growth were reported by other authors who showed enhanced wheat grains germination and seedlings growth after priming the seeds with ZnONPs at a concentration of 10 mg L^-1^ (Rai-Kalal and Jajoo, 2021). Recently, the mechanisms of ZnONPs action as a nanobiofeltilizer for plant growth promotion were studied by Sun et al. (2020), Khan et al. (2021) and El-Badri et al. (2021). They found that these NPs increased zinc uptake by plants, maximized plant production and improved plant resistance to biotic and abiotic stresses. In turn, Itroutwar and coworkers (2020a) identified the accumulation of ZnONPs in maize seeds endosperm leading to improved germination. It has been suggested that ZnONPs facilitate water uptake and increase α-amylase activity during germination (Itroutwar et al., 2020b; Rai-Kalal and Jajoo, 2021; Sharma et al., 2021). Moreover, study conducted by El-Badri and coworkers (2021) showed that ZnONPs activity as plant growth promoter was associated with increasing metabolic activity and modulating the expression of hormone genes (abscisic acid (ABA) and gibberellic acid (GA)) during seed germination. In another study, Rawashdeh et al. (2020) showed improved germination and enhanced biomass production under salt stress after nanopriming seeds with ZnONPs. The mechanism of action of ZnONPs was attributed to stimulation of enzyme biosynthesis, induction of carbohydrate decomposition and increased activity of the antioxidant system.

Both positive and negative effects of ZnONPs on seed germination and plant growth have been reported, but many studies have shown that bio-synthesized ZnONPs are more stable and biocompatible (due to capping and stabilizing agents of natural origin on their surface) compared to chemical ones (Singh et al., 2018). The use of bio-ZnONPs in the preparation of seeds of agronomically important crops can contribute to increased crop productivity and quality. Although there is still limited information on the interaction of nanoparticles with plants, further efforts should be made to clarify them (Tondey et al., 2021).

## 5 Conclusion

In this study, AgNPs and ZnONPs were effectively synthesized from *F. solani* IOR 825. They were comprehensively characterized using UV–vis, TEM, XRD, DLS, Zeta potential measurements, and FTIR which revealed the small size and spherical shape of AgNPs, the larger size of ZnONPs, and for both stability, crystalline nature, and that mycosynthesized nanoparticles were capped with biomolecules. The AgNPs were found to have strong antimicrobial potential against bacterial pathogens of plants*.* The lower sensitivity of pathogenic bacteria was demonstrated to ZnONPs. In addition, both types of NPs showed the potential to inhibit fungal spore germination, which is crucial in the fungal spread in the environment, and growth of fungal mycelia. The AgNPs revealed sterilization effect on maize seeds while ZnONPs demonstrated stimulatory effect on seedlings growth by improving fresh and dry biomass production. The present work highlights that mycosynthesized silver and zinc oxide nanoparticles in view of their unique properties, have a high potential as a promising agent to control or prevent the growth of pathogens in agriculture and enhance crop productivity. Nevertheless, continued investigations into their effects on plant development, growth and long-term toxicity are required.

## Data Availability

The original contributions presented in the study are included in the article/Supplementary Material, further inquiries can be directed to the corresponding authors.
