# Supplementary material for: Biofabrication of novel silver and zinc oxide nanoparticles from Fusarium solani IOR 825 and their potential application in agriculture as biocontrol agents of phytopathogens, and seed germination and seedling growth promoters
Source: Front Chem. 2023 Aug 4;11:1235437. doi: 10.3389/fchem.2023.1235437 (PMC10436318; doi:10.3389/fchem.2023.1235437)
Supplement: Supplementary file 1 [file DataSheet1.pdf]

Supplementary Material

Biofabrication of novel silver and zinc oxide nanoparticles from *Fusarium solani* IOR 825 and their potential application in agriculture as biocontrol agents of phytopathogens, and seed germination and seedling growth promoters

Joanna Trzcińska-Wencel [1*], Magdalena Wypij [1], Artur P. Terzyk[2], Mahendra Rai [1,3], Patrycja Golińska[1*]

[1] Department of Microbiology, Faculty of Biological and Veterinary Sciences, Nicolaus Copernicus University in Toruń, Toruń, Poland

[2] Physicochemistry of Carbon Materials Research Group, Department of Chemistry of Materials, Adsorption and Catalysis, Faculty of Chemistry, Nicolaus Copernicus University in Toruń, Toruń, Poland

[2]Nanobiotechnology Laboratory, Department of Biotechnology, SGB Amravati University, Amravati, India;

*** Correspondence:**
Joanna Trzcińska-Wencel
trzcinska@doktorant.umk.pl
Patrycja Golińska
golinska@umk.pl

Supplementary Table 1. Maximum adsorption peaks of mycosynthesized AgNPs and ZnONPs from UV-vis analysis and synthesis efficiency expressed as mg of NPs per 100 mL of *F. solani* IOR 825 extract.

	AgNPs	ZnONPs (1)	ZnONPs (2)
A_{max} wavelength [nm]	420	357	357
Synthesis yield [mg/100 ml]	26.35	435.56	525.8

ZnONP (1); first method of nanoparticle synthesis, ZnONP (2); second method of nanoparticle synthesis

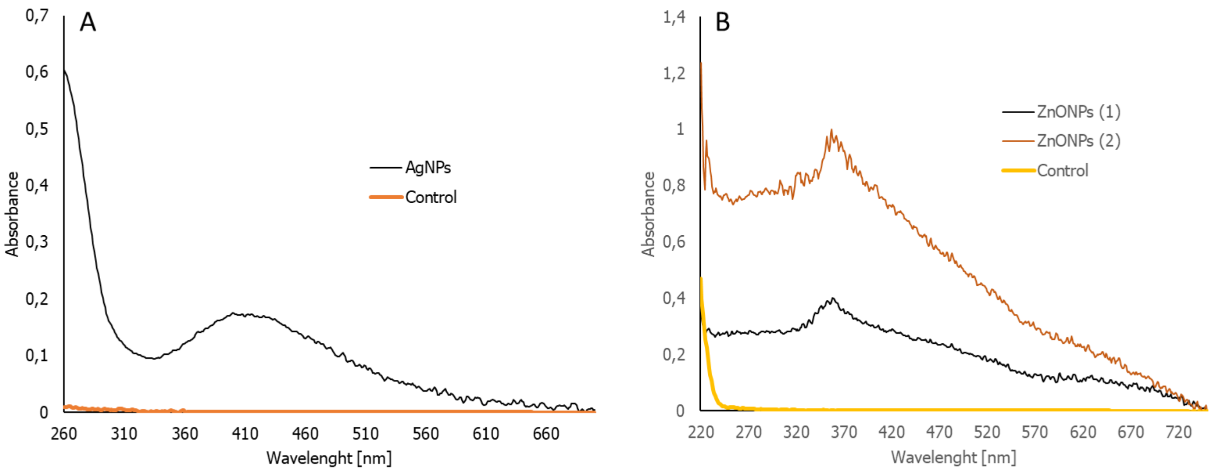

Supplementary Figure 1. UV-vis spectra of AgNPs (A) and ZnONPs (B) synthesized from *Fusarium solani* IOR 825.
ZnONP (1); first method of nanoparticle synthesis, ZnONP (2); second method of nanoparticle synthesis

Supplementary Table 2. Elemental composition of nanoparticles from *Fusarium solani* IOR 825 based on EDX analysis [weight %].

Element	AgNPs	ZnONPs (1)	ZnONPs (2)
Ag	55.43	-	-
Zn	-	70.94	78.67
O	-	18.76	19.35
other	44.56 (C)	10.03 (Mo, Al, Si)	1.98 (C, Al)

ZnONP (1); first method of nanoparticle synthesis, ZnONP (2); second method of nanoparticle synthesis
